# Time-Restricted Eating and Bone Health: A Systematic Review with Meta-Analysis

**DOI:** 10.3390/nu16060876

**Published:** 2024-03-18

**Authors:** Rubén Fernández-Rodríguez, Miriam Garrido-Miguel, Bruno Bizzozero-Peroni, Valentina Díaz-Goñi, Eva Rodríguez-Gutiérrez, María José Guzmán-Pavón, Ana Belén Meseguer-Henarejos, Ana Torres-Costoso

**Affiliations:** 1 Health and Social Research Center, Universidad de Castilla-La Mancha, 16002 Cuenca, Spain; ruben.fernandez@uclm.es (R.F.-R.); miriam.garrido@uclm.es (M.G.-M.); valentina.diaz@uclm.es (V.D.-G.); eva.rodriguez@uclm.es (E.R.-G.); anaisabel.torres@uclm.es (A.T.-C.); 2 Research Network on Chronicity, Primary Care and Health Promotion (RICAPPS), 16002 Cuenca, Spain; 3 Faculty of Nursing, Universidad de Castilla-La Mancha, 02006 Albacete, Spain; 4 Instituto Superior de Educación Física, Universidad de la República, Rivera 40000, Uruguay; 5 Faculty of Physiotherapy and Nursing, Universidad de Castilla-La Mancha, 45071 Toledo, Spain; mariajose.guzman@uclm.es; 6 Faculty of Medicine, Department of Physiotherapy, Universidad de Murcia, 30120 Murcia, Spain; anabelen@um.es

**Keywords:** time-restricted eating, diet, bone mineral density, bone mineral content, bone turnover marker, systematic review, meta-analysis

## Abstract

Time-restricted eating (TRE) has emerged as a dietary strategy that restricts food consumption to a specific time window and is commonly applied to facilitate weight loss. The benefits of TRE on adipose tissue have been evidenced in human trials and animal models; however, its impact on bone tissue remains unclear. To systematically synthesize and examine the evidence on the impact of TRE on bone health (bone mineral content (BMC), bone mineral density (BMD), and bone turnover factors), PubMed, Scopus, Cochrane CENTRAL, and Web of Science databases were systematically explored from inception to 1 October 2023 searching for randomized controlled trials (RCTs) aimed at determining the effects of TRE on bone health in adults (≥18 years). The Cochrane Handbook and the PRISMA recommendations were followed. A total of seven RCTs involving 313 participants (19 to 68 years) were included, with an average length of 10.5 weeks (range: 4 to 24 weeks). Despite the significant weight loss reported in five out of seven studies when compared to the control, our meta-analysis showed no significant difference in BMD (g/cm^2^) between groups (MD = −0.009, 95% CI: −0.026 to 0.009, *p* = 0.328; *I*^2^ = 0%). BMC and bone turnover markers between TRE interventions and control conditions were not meta-analyzed because of scarcity of studies (less than five). Despite its short-term benefits on cardiometabolic health, TRE did not show detrimental effects on bone health outcomes compared to those in the control group. Nevertheless, caution should be taken when interpreting our results due to the scarcity of RCTs adequately powered to assess changes in bone outcomes.

## 1. Introduction

Bone health is critically important because of the skeleton and its function of support and protection. Bones are dynamic tissues (bone remodeling throughout the lifespan) that might play an endocrine role [1,2]. Bones are closely related to energy metabolism [3] through various hormones such as osteocalcin, and they maintain a cross-talk interaction with muscles through different secretory factors [2]. For instance, energy uptake impacts bone biology since a lack of energy intake results in a loss of bone mass. Therefore, weight loss has been associated with a detrimental effect on bone health [4]. There are several factors underlying the impact of weight loss on bone health, including clinical, medical, behavioral, nutritional, and genetic variables (i.e., reduced mechanical load, loss of muscle mass, and changes in the secretion of gut hormones and adipokines) [5,6]. Indeed, nutritional aspects, including dietary patterns and strategies are among the modifiable lifestyle factors related to bone health [7,8,9]. Some studies showed that people with overweight and obesity have greater bone mineral density (BMD) when compared to individuals with normal weight [10,11,12,13]. Contrarily, other authors have suggested that people with overweight or obesity have worse bone quality than their peers due to the metabolic impact of excess adipose tissue [14,15].

Regarding weight loss interventions based on dietary strategies, caloric restriction and intermittent fasting (IF) are common approaches to reducing energy intake that have shown effectiveness on weight loss and cardiometabolic-related outcomes [16]. Despite this, epidemiological evidence that considers breakfast omission as IF is associated with bone loss [17], and caloric restriction alone or in combination with exercise has been shown to reduce bone mass and negatively affect bone microstructure [18,19,20,21]. These negative effects could be caused by mechanical unloading, nutrient deficiencies, and endocrine changes caused by a low energy intake [22,23]. Nevertheless, a common IF protocol, alternate day fasting (ADF), has not shown changes in total bone mineral content (BMC) or BMD after achieving significant weight loss [24,25].

Accordingly, a dietary strategy based on chrono nutrition, time-restricted eating (TRE), has emerged as a feasible and safe weight loss intervention. TRE has shown cardiometabolic benefits [26] with possibly higher acceptability and compliance [27] than other IF or caloric restriction interventions due to its simplicity and ease of implementation [28]. TRE is a daily IF approach that involves consuming all calories within a window of ≤12 h [29]. TRE might reduce possible detrimental effects by synchronizing eating behaviors with endogenous circadian rhythms that align with metabolic control [30,31,32] and may, ultimately, benefit bone health. Bone tissue is sensitive to circadian rhythmicity [32]; thus, realignment of meal timing with circadian rhythms through TRE might promote bone preservation independently of weight. The largest study of TRE assessing bone outcomes (6-month intervention) showed that when weight loss occurs, TRE might be associated with some bone-sparing effects compared with standard dietary advice [33]. However, we should be aware that the study by Papageorgiou et al. [33] applied a mildly TRE (12 h eating window), most participants were women (76%) in menopausal status age (median of 47 years), and one in three individuals had obesity or metabolic syndrome.

Consequently, despite the well-established benefits of TRE in cardiometabolic parameters, the effects of TRE protocols on bone health are far from conclusive. Therefore, our systematic review and meta-analysis aimed to synthesize the available evidence and determine the effects of TRE on bone health (i.e., BMC, BMD, and bone turnover markers) in the general adult population.

## 2. Materials and Methods

The guidelines of the *Cochrane Collaboration Handbook* [34] and the Preferred Reporting Items for Systematic Reviews and Meta-Analyses (PRISMA) [35] were followed. The systematic review protocol was previously registered in the PROSPERO database (reference number *CRD42023463012*).

### 2.1. Data Sources and Search Strategy

Two independent reviewers (RFR and ATC) systematically examined PubMed, Scopus, Cochrane CENTRAL, and Web of Science from inception to 1 October 2023. The rationale for the search strategy was performed using the patient, intervention, comparison, outcome, and study design (PICO) approach. Keywords such as “time-restricted eating”, “time-restricted feeding”, “bone”, or “bone mineral density” comprised the search strategy aimed at identifying randomized controlled trials (RCTs) analyzing the effects of TRE on bone health in the general adult population. The EndNote software (Endnote 20.6) was used for the screening process, and a third coauthor peer-reviewed the entire search progression (BBP). The complete search strategy for each database is detailed in Table S1.

### 2.2. Eligibility Criteria

The PICOs framework for study selection was as follows: (i) participants: adults (age average ≥18 years); (ii) intervention: different protocols of TRE intervention; (iii) comparator: non-TRE conditions (e.g., habitual diet and standard dietary advice); (iv) outcomes: parameters related to bone health (i.e., BMC, BMD, and bone turnovers markers); and (v) study design: RCTs. No language restriction was applied. We did not consider those RCTs in which TRE was applied with more than 12 h of an eating window. No additional exclusion criteria were applied. Studies excluded after full-text reading with reasons are available in Table S2.

### 2.3. Data Collection

Two reviewers (RFR and VDG) independently extracted the following information from each trial: (1) first author name, year of publication, and country; (2) sample characteristics (i.e., health status, sample size, % female, mean age, and body mass index (BMI)); (3) characteristics related to TRE interventions (i.e., type, fasting window duration, food consumption schedules, and length in weeks) and comparison groups (i.e., type); (4) outcomes: parameters related to bone health and standardized assessment methods used to assess them; and (5) main results related to bone and body composition. A third coauthor (BBP) independently assessed the accuracy of the extracted data.

### 2.4. Quality and Certainty Assessment

The risk of bias of the included RCTs and the certainty of the evidence was assessed independently by two authors (ERG and MGM). A third author (MJGP) was consulted in case of disagreement. The risk of bias in the RCTs was assessed using the Cochrane Collaboration tool for assessing the risk of bias (RoB2) [36]. The RoB2 tool evaluated the risk of bias according to five domains: (i) randomization process, (ii) deviations from intended interventions, (iii) missing outcome data, (iv) measurement of the outcome, and (v) selection of the reported result. Overall bias was scored as (i) “low risk of bias” if all the domains of the study were classified as “low risk”, (ii) “some concerns” if at least one domain was scored as “some concerns”, and (iii) “high risk” if at least one domain was scored as “high risk” or several domains as “some concerns” and could affect the validity of the results. The Risk-of-bias VISualization (robvis) tool was used to develop the figures for the risk of bias assessments [37].

The certainty of the evidence was determined according to the “Grades of Recommendations, Assessment, Development, and Evaluation” (GRADE) tool [38]. Based on study design, risk of bias, indirect evidence, inconsistency, publication bias, and imprecision, the outcome was judged as high-, moderate-, low-, or very low-quality evidence.

### 2.5. Data Synthesis

A meta-analysis was conducted when a minimum of five studies addressed the same outcome (i.e., for BMD) [39]. When a quantitative synthesis was not possible, the bone health data of the included studies were synthesized narratively (i.e., for BMC and bone turnover markers). As significant between-study heterogeneity was anticipated, a random-effects method was used to pool the difference in means between TRE interventions vs. non-TRE groups on BMD. The DerSimonian and Laird method was applied to estimate the heterogeneity variance [40]. Heterogeneity across the included studies was assessed using the *I*^2^ metric, classified as not important (0–40%), moderate (30–60%), substantial (50–90%), or considerable (75–100%), and corresponding *p*-values were also considered [34]. All analyses were conducted using R software (version 4.2.3; R Foundation for Statistical Computing) with the meta package [41].

#### 2.5.1. Measure of Intervention Effect

Between-group mean differences (MDs) and their 95% confidence intervals (CIs) were calculated for each study. The unstandardized difference in means between two independent groups (i.e., TRE interventions vs. non-TRE conditions) was used since all studies measured BMD on the same scale (i.e., g/cm^2^). Positive MD values favored TRE interventions, and negative MD values favored control conditions in pre-post BMD changes following trial interventions.

#### 2.5.2. Outcome Data for Evidence Synthesis

For all parameters of bone health that were synthesized narratively (i.e., BMC and bone turnover markers), baseline and post-intervention outcome data (mean and standard error (SE)) were extracted. For the meta-analysis of BMD, the pre-post MD and their standard deviation (SD) within TRE interventions and control conditions were extracted to estimate the between-group MD. Additionally, a supplementary meta-analysis for lean mass and fat mass was conducted to determine the effects of TRE vs. control conditions.

#### 2.5.3. Sensitivity Analyses

Sensitivity analyses were carried out to assess the robustness of the summary estimates via the leave-one-out method [34]. Furthermore, additional analyses were conducted while upholding the prevailing health status of the participants included in the RCTs (i.e., overweight and/or obesity) [42,43,44,45] and the predominant TRE protocol (i.e., fasting window 16:8) [42,43,44,46].

### 2.6. Other Metodological Considerations

When RCTs reported results for different parameters of bone health [42,44,45,47], data were included according to the appropriate analysis. Where between-group MDs were displayed only graphically [43], data were extracted using online software (WebPlotDigitizer, assessed on 2 December 2023) [48]. Furthermore, in a study that reported pre-post changes in bone health outcomes as medians and interquartile ranges [45], the method of McGrath et al., 2020 [49] was applied to estimate the means and SDs. In those cases where trials reported effect estimates with their SEs or CIs, SDs for meta-analysis were calculated using the following formulas: (i) SD = √n (SE), and (ii) SE = (upper limit of CI − lower limit of CI)/3.92 [34]. Finally, in a RCT that reported results among four independent groups, i.e., TRE vs. non-TRE and TRE plus exercise vs. non-TRE plus exercise [43], these were considered two independent comparisons for quantitative analyses.

## 3. Results

### 3.1. Literature Search

**Figure 1:**
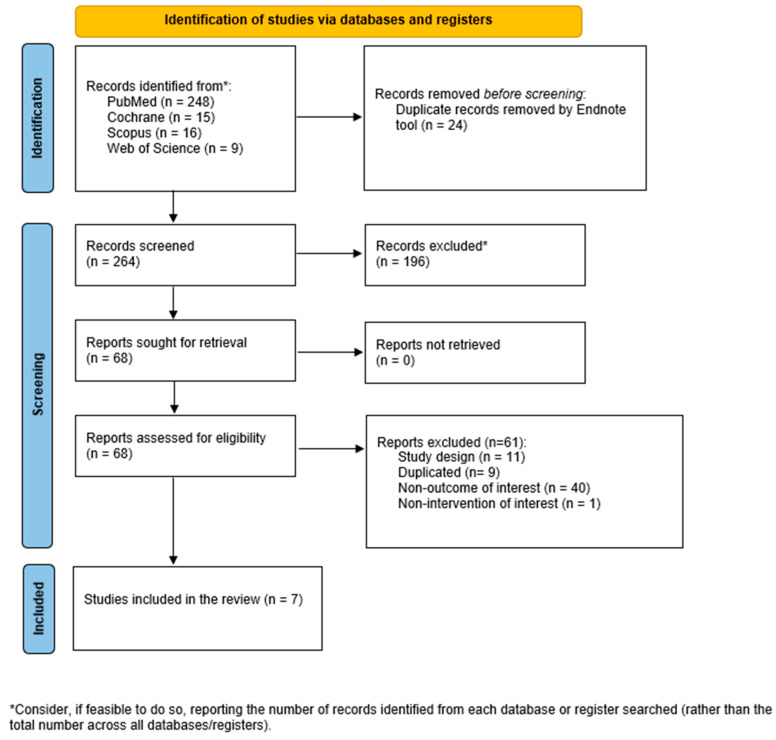
Flowchart of the studies through the review (PRISMA 2020).

A total of 264 studies were considered for title-abstract review after removing duplicates, of which 68 were fully assessed for eligibility, and 61 were excluded for the reasons described in Table S2. Finally, seven RCTs were included in this systematic review and meta-analysis (Figure 1). Among them, 14 comparisons (4 for BMC, 6 for BMD, and 4 for bone turnover markers) between TRE interventions vs. control conditions were included.

### 3.2. Study Characteristics

Table 1 summarizes the main characteristics of the included studies. The RCTs were designed as parallel [42,43,44,45,47] and crossover [46,50] studies and were conducted between 2020 and 2023 in three different countries (i.e., USA, China, and Switzerland).

### 3.3. Population

The included studies comprised a total of 313 adults (203 females and 110 males), with a mean age range between 20.3 to 66.0 years. The trials included healthy individuals [46,50] or participants with specific conditions, including overweight and/or obesity [42,43,44,47] and metabolic syndrome [45]. The mean BMI ranged from 21.6 to 33.8 kg/m^2^ in the TRE interventions and from 20.3 to 34.4 kg/m^2^ in the control groups.

### 3.4. Time-Restricted Eating Interventions

The mean length of the interventions was 10.5 weeks, ranging between 4 and 24 weeks. TRE interventions were defined as self-selected [44,45,46,50] or according to the application of specific recommendations and instructions for the timing of food intake [42,43,47]. The 16:8 TRE protocol was the most reported in the included RCTs, except for Papageorgiou et al., 2023, which was 12:12 [45]. Table 1 shows the dosage of the interventions in terms of food consumption schedules.

### 3.5. Control Conditions

The main control condition applied via the RCTs was to follow usual eating habits and patterns [42,43,44,45,46,50]. Two RCTs applied standard dietary instructions or advice for the non-TRE groups [45,47].

### 3.6. Bone Health Outcomes

Bone mineral density and BMC were assessed using dual-energy X-ray absorptiometry techniques in six [42,43,44,45,46,50] and four [42,44,45,47] studies, respectively. Five trials reported data on total body BMD [42,43,44,45,46] and one trial on regional BMD (i.e., head, arms, ribs, spine, trunk, pelvis, and legs) [50]. Furthermore, two RCTs [44,45] analyzed different bone turnover markers related to bone formation (the N-terminal propeptide of type I procollagen [P1NP]) and bone resorption (C-terminal telopeptide of type I collagen [CTX] and the N-terminal telopeptide of type I collagen [NTX]).

### 3.7. Comparisons Not Included in the Meta-Analysis

Tables S3 and S4 summarize the main results of the included studies. Due to the small number of RCTs (n < 5), MDs for BMC and bone turnover markers between TRE interventions and control conditions were not meta-analyzed. Studies have provided mixed results for BMC in middle-aged adults after trial interventions (Table S3). Participants showed an increase in total body BMC after TRE interventions and a greater change compared to control groups in two RCTs, although no significant differences were observed [44,47]. In turn, two RCTs showed a decrease in total body BMC after the trial interventions, with greater reductions for both the TRE [42] and non-TRE [45] groups. Furthermore, there were no significant within-group and between-group differences in two studies [44,45] that analyzed bone turnover markers (i.e., CTX, P1NP, and NTX) in middle-aged adults (Table S4).

### 3.8. Meta-Analysis

**Figure 2:**
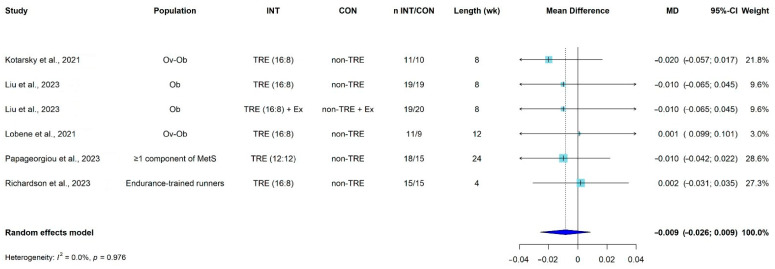
Pooled mean difference for the effect of time-restricted eating interventions vs. control groups on bone mineral density (g/cm^2^) in young and middle-aged adults. Abbreviations: BMD, bone mineral density; CI, confidence interval; CON, control group; Ex, exercise; INT, intervention; MD, mean difference; MetS, metabolic syndrome; Ob, obesity; Ov, overweight; TRE, time-restricted eating; wk, weeks [42,43,44,45,46].

The meta-analysis included six comparison groups between TRE interventions and non-TRE groups in young and middle-aged adults. A non-significant MD in total body BMD was observed when comparing the TRE interventions (n = 93 participants) with the non-TRE groups (n = 88 participants) after trial intervention periods between 4 to 24 weeks. Specifically, there was no significant difference in BMD (g/cm^2^) between groups (MD = −0.009, 95% CI: −0.026 to 0.009, *p* = 0.328; *I*^2^ = 0%) (Figure 2, Table S5). All the studies included in the meta-analysis reported significant weight loss when TRE interventions were compared to control conditions [42,43,44,45,46]. Furthermore, there were non-significant MD on fat mass when comparing TRE interventions (n = 99 participants) with non-TRE groups (n = 95) (MD = −0.56, 95% CI: −1.40 to 0.29; *I*^2^ = 0%) [42,43,44,46,47], neither on lean mass (TRE interventions = 84 participants vs. non-TRE groups = 80 participants) (MD = −0.76, 95% CI: −1.61 to 0.10, *I*^2^ = 0%) [42,43,44,47] between groups (Tables S6 and S7).

#### 3.8.1. Sensitivity Analyses

The results of the sensitivity analyses were consistent with the main results. The pooled MD was not modified when each study in RCTs was removed one by one to examine the effect of TRE interventions vs. control conditions on BMD (Table S8). In turn, MDs between TRE interventions and non-TRE groups for total body BMD remained non-significant after maintaining both the health status (i.e., overweight and/or obesity) and TRE protocol (i.e., 16:8) predominant in the included studies (Table S9). Specifically, there was no significant difference in BMD (g/cm^2^) between groups in RCTs that analyzed only overweight and/or obese participants (MD = −0.013, 95% CI: −0.033 to 0.008, *p* = 0.223; *I*^2^ = 0%) and implemented only 16:8 TRE protocols (MD = −0.008, 95% CI: −0.028 to 0.012, *p* = 0.441; *I*^2^ = 0%).

#### 3.8.2. Risk of Bias and Certainty Assessment

The overall risk of bias, assessed by the RoB2 tool, was scored as “some concerns” for all the studies (mainly related to the selection of the reported results) (Figure S1). According to the GRADE approach, the quality of the evidence of the effect of TRE interventions on BMD was “low” since the certainty assessment showed serious concerns regarding the risk of bias and imprecision. A table summarizing the findings is available in Table S10.

## 4. Discussion

Despite the significant weight loss of TRE compared to control conditions, our data suggest that TRE does not harm bone health. TRE did not show a significant reduction in total body BMD. Moreover, our results were consistent when considering only people with overweight and obesity and TRE 16:8 protocols. Although fat mass decreased in all TRE groups while increased in three out of six control conditions, there were non-significant differences between groups, and more importantly, lean mass did not decrease significantly. Consequently, TRE might be recommended as a dietary strategy for weight loss in the short term (up to 6 months) without concerns related to bone health. Nevertheless, caution should be taken when interpreting our results due to the scarcity of RCTs adequately powered to assess changes in bone outcomes, the short length of most included studies, and the lack of detailed information regarding lifestyle factors (i.e., smoking, alcohol consumption, diet quality, physical activity, sleeping patterns, etc.) that might influence bone health.

Studies included in our review are in line with previous evidence [26,51,52,53,54] showing the beneficial effects of TRE interventions on body weight and fat mass. Considering this, the most remarkable finding of the present review is that there were no harmful effects on bone health markers despite the reduction in body weight and fat mass. Our review (n = 313) showed that most individuals assigned to TRE interventions achieved significant weight loss and positive changes in body composition related to decreased fat mass, percentage of body fat, and visceral fat compared to those in the pre-TRE interventions and non-TRE groups [42,43,44,45,46]. Additionally, there were only two studies in which the significant weight loss was only intragroup for TRE [47,50], one of them a crossover-designed RCT in which participants assigned to TRE maintained their body mass throughout the intervention [50]. Moreover, we should be cautious when interpreting the results of the study by Lowe et al., 2020 [47] because more than half of the weight loss was fat-free mass, and participants reported reduced protein intake; thus, this could affect their findings. Finally, studies reporting lean mass showed a non-significant reduction in lean mass [43,44,47], except for Kotarsky et al. (2021) [42], who showed a maintained lean mass, maybe due to participants performing concurrent training. Despite this, they did not find an effect of the intervention on bone health, probably because although lean mass is an important determinant of bone health because it is an excellent marker of mechanical bone stimulation [13], the relationship between fat mass and lean mass in complex and favorable metabolic changes associated with the decrease in fat mass [55] may compensate for the damage caused by the reduction of lean mass, although other nutritional and environmental factors should be considered.

In contrast with our findings, other dietary strategies for weight loss have been related to detrimental effects on bone health (i.e., changes in bone turnover markers and reductions in BMD/BMC) [56,57,58]. Consequently, TRE might emerge as a potential approach for weight loss without a negative impact on bone health at least in the short term. Nevertheless, the heterogeneity shown by the population (aged 20.3 to 66.0 years) and the baseline bone health of individuals included (i.e., post-menopausal and older adults) might be considered when extrapolating these results. Included studies in our additional meta-analyses for fat and lean mass did not show significant reduction when comparing TRE vs. control conditions, although all TRE interventions decreased fat mass. Considering this and the crucial role of lean mass for bone health, it would be recommended that future TRE protocols include a resistance training program and adequate and balanced dietary patterns added to individualized advice in populations at high risk for bone fragility to maximize its benefits.

In the last decade, TRE has increased in popularity because of its beneficial effects on weight loss and simplicity (no need to count calorie intake or food restrictions). Currently, weight loss ranges from 5 to 10% (1–3 months), and TRE is often associated with benefits in cardiometabolic health despite the lack of weight change [52,53]. However, long-term studies (12 months) demonstrated a 5% reduction in body weight in people with obesity, which could be extrapolated as a “plateau” effect after the first 4 to 12 weeks [52]. As with TRE for bone health endpoints, studies exploring the long-term effects of TRE interventions are lacking, and it is expected that in the coming years, evidence about its impact could lead to more solid conclusions. TRE interventions might induce changes in bone metabolism and health through different mechanisms. For instance, TRE interventions could result in detrimental or neutral effects on bone health due to weight loss [18], changes in body composition [59], endocrine profile or hormonal concentrations, and some lifestyle behaviors [31,54,60]. Conversely, TRE could be related to positive changes in the gut microbiome, inflammatory biomarkers, and oxidative stress that may positively impact bone health [61,62]. Moreover, the realignment with the circadian rhythm might positively affect bone metabolism [32]. Nevertheless, as mentioned above, further controlled RCTs need to unravel the scientific evidence for the intrinsic mechanism that might influence bone health during a TRE intervention.

Our review has some limitations that should be noted. First, only a few studies were included (n = 7) with small sample sizes in each arm trial (from 9 to 59 participants), and our meta-analysis compared a total of 93 individuals assigned to TRE interventions vs. 88 individuals in non-TRE comparison groups. This, added to most studies, did not explore bone parameters as the primary outcome raises some concerns about whether the studies included were adequately powered to detect statistically significant differences in bone health. Second, it is well-known that bone remodeling occurs in periods of about 6 months, and most studies were short-term (4–12 weeks), with only one RCT and a long follow-up (6 months) [45]. In fact, this limitation should be carefully considered. To date, TRE could be implemented as a dietary strategy in the short and medium term (i.e., 6 months), but future studies should assess the effects of TRE on the physiology and/or pathophysiology of bone tissue. Finally, despite our stratified analysis considering only people with overweight or obesity and metabolic disorders, the heterogeneity among the population included may challenge the extrapolation of our results.

In summary, our findings suggest that TRE did not have detrimental effects on bone health despite the reduction in body weight and fat mass. Nevertheless, the current evidence is limited. Consequently, it is necessary to conduct further RCTs with a larger sample size that should focus on people at risk for bone fragility (i.e., post-menopausal women) in the long term (≥ 6 months). These studies should be adequately powered to determine changes in bone outcomes and designed to include clinically relevant bone assessment (i.e., BMD at the hip/lumbar spine, BMC, and bone microstructure), including bone turnover markers that can be used to predict changes in short-term trials (<6 months). This would aid in establishing solid conclusions.

## Figures and Tables

**Table 1:** Main characteristics of studies included in the systematic review.

Author Year Country	Sample Characteristics *	TRE Protocol	Length (Weeks)	Comparator	BMD/BMC Assessment	Outcomes	Results
Lowe et al., 2020 [47] USA	Overweight and obese adults aged 18–64 y N (% female):116 (40.7%) TRE: 59; CON: 57 Age (y): RE: 46.8 ± 10.8; CON: 46.1 ± 10.3 BMI(kg/m^2^): TRE = 32.9 ± 4.9/CON = 32.6 ± 3.4	16:8 (eating window from 12 pm to 8 pm)	12	non-TRE (CON)	DXA: Hologic Horizon/A system (Hologic Inc., Marlborough, MA, USA).	BMC total	Weight: Significant weight loss in TRE but not significant between-groups differences compared to CON. Bone: No significant increment on BMC.
Martens et al., 2020 [50] USA	Healthy non-obese midlife and older adults N (% female): 22 (54.5%) TRE:12; CON: 10 Age (y): TRE: 66.0 ± 2.0; CON: 68.0 ± 2.0 BMI (kg/m^2^): GA (Non-TRE, TRE) = 25.7 ± 0.7/GB (TRE, Non-TRE) = 23.9 ± 0.9	16:8 (self-selected, eating window from 10–11 am to 6–7 pm)	6	non-TRE (CON)	DXA: Lunar/Prodigy, GE Healthcare, Chicago IL, USA.	BMD (total and regional)	Weight: Body weight maintenance-throughout the TRE intervention (Non-TRE 69.3 ± 2.7 vs. TRF 69.4 ± 2.8 kg; *p* = 0.82). Bone: No change in BMD between-groups. Total and regional BMD were not different between conditions.
Kotarsky et al., 2021 [42] USA	Physically inactive and overweight or obese N (% female): 21 (85.7%) TRE: 11; CON: 10 Age (y): TRE: 45 ± 3; CON: 44 ± 2 BMI (kg/m^2^): TRE= 29.8 ± 0.8/CON = 29.4 ± 0.8	16:8 (eating window from 12 pm to 8 pm)	8	non-TRE (CON), both groups were performing concurrent training	DXA: on a Lunar Prodigy, Model #8915 (GE Healthcare).	BMD, BMC (total)	Weight: Losses of total body mass were significantly greater for TRE (3.3%) relative to NE (0.2%) pre-to post-intervention, of which TRE had significantly greater losses of fat mass (9.0%) compared to CON (3.3%). Lean mass increased during the intervention for both TRE (0.6%) and CON (1.9%), with no group differences. Bone: No significant change or differences between-groups.
Lobene et al., 2021 [44] USA	Overweight and obese adults aged 18–65 y N (% female): 20 (85%) TRE: 11; CON: 9 Age (y): TRE: 46.5 ± 3.7; CON: 44.2 ± 4.1 BMI (kg/m^2^): TRE: 33.8 ± 2.3; CON: 34.4 ± 2.6	16:8 (self-selected)	12	non-TRE (CON)	DXA: scans using the enCore software (GE Healthcare, Chicago, IL, USA, version 16.2).	BMD total and bone turnover (P1NP, NTX, PTH)	Weight: Body weight, fat mass, lean mass, and visceral fat were reduced in the TRE group compared to pre-intervention (−3.7% ± 0.5; −4.0% ± 0.9; −3.0 ± 0.8, and −11.1% ± 4.0, respectively), and changes in body weight, lean mass, and visceral fat were significant compared to the non-TRE group (all *p* < 0.05). Bone: No significant treatment effects on bone health outcomes
Liu et al., 2023 [43] China	Female college students with hidden obesity N (% female): 77 (100%); TRE: 19; CON: 19; EX: 20; TRE + EX: 19 Age (y): TRE: 20.3 ± 1.8; CON: 20.1 ± 1.8; EX: 20.1 ± 1.4; TRE + EX: 19.9 ± 0.6 BMI (kg/m^2^): TRE = 21.63 ± 1.24/CON = 20.32 ± 1.06	16:8 (eating window from 10 am to 6 pm)	8	Control, EX and TRE + EX	DXA: Hologic, Horizon, WI, USA.	BMD (total)	Weight: Significant weight loss, BMI, lean tissue mass on TRE. Bone: Total BMD (TRE, EX, and TRE + EX) and the CON group showed no significant differences (*p* > 0.05).
Richardson et al., 2023 [46] USA	Long-distance male runners N (% female): 15 (0%) Age (y): 28.7 ± 5.2 BMI (kg/m^2^): 23.3 (calculated from primary data on weight and height)	16:8 (self-selected)	4	non-TRE (12 h eating window) 4 weeks intervention Wash-out: 2 to 4 weeks	DXA: Hologic Discovery QDR Series 94994; Hologic, Inc.	BMD total, BMD z-score	Weight: Significantly losses of fat mass, leg fat mass, and percent body fat in the TRE intervention, with no change in fat-free mass. Bone: No change.
Papageorgiou et al., 2023 [45] Switzerland	Adults with ≥1 component of metabolic syndrome N (% female): 42 (76%); TRE: 23; CON: 19 Age (y): TRE: 47 (range: 32–57); CON: 45 (range: 27–50) BMI (kg/m^2^): TRE = 28.51 ± 4.47/CON = 27.37 ± 5.18	12:12 (self-selected)	24	non-TRE (SDA)	DXA: GE Healthcare Lunar iDXA at Lausanne site, GE Healthcare Lunar Prodigy Advance at Bern site.	BMD/BMC (total), and bone turnover markers (P1NP, NTX, PTH, CTX, vit D, IGF-1)	Weight: Participants significantly lost weight after 6 months of TRE. Bone: No overall detrimental effects of 6 months of TRE on bone health outcomes. Those who lost weight following the CON intervention (SDA) experienced small, albeit non-significant, increases in CTX levels without parallel changes in P1NP levels and a small loss of total body BMC. Weight loss responders with TRE tended to have reduced bone resorption (CTX) whereas no change occurred in bone formation (P1NP). As opposed to the bone loss observed in weight loss responders with SDA, total body BMC/BMD remained unaltered in weight loss responders after TRE.

*: Age and BMI reported with mean ± standard deviation or mean (range). **Abbreviations: TRE**, time-restricted eating; **y**, year; **CON**, control group; **BMI**, body mass index; **BMD**, bone mineral density; **BMC**, bone mineral content; **EX**, exercise; **P1NP**, procollagen type 1 N-terminal propeptide; **NTX**, cross-linked N-telopeptide of type I collagen; **PTH**, parathyroid hormone; **CTX**, serum β-carboxyterminal telopeptide of type I collagen; **vit D**, total 25-hydroxyvitamin D; **IGF-1**, insulinlike growth factor 1; **SDA**, standard dietary advice; **DXA**: dual-energy X-ray absorptiometry.

## Data Availability

Data will be available upon request to the corresponding author.

## Supplementary Materials

The following supporting information can be downloaded at: https://www.mdpi.com/article/10.3390/nu16060876/s1, Table S1: Search strategy for each database; Table S2: Reason for exclusion after full-text screening (n = 61); Table S3: Effects of time-restricted eating interventions on bone mineral content compared to control conditions; Table S4: Effects of time-restricted eating interventions on bone turnover markers compared to control conditions; Table S5: Effects of time-restricted eating interventions on bone mineral density compared to control conditions; Table S6: Effects of time-restricted eating interventions on fat mass compared to control conditions; Table S7: Effects of time-restricted eating interventions on lean mass compared to control conditions; Table S8: Sensitivity analyses using the leave-one-out method; Table S9: Sensitivity analysis after maintaining the predominant health status and time-restricted eating protocol among the included studies; Table S10: Quality of evidence assessment according to the GRADE approach; Figure S1: Risk of bias assessment [42,43,44,45,46,47,48,49,50,63,64,65,66,67,68,69,70,71,72,73,74,75,76,77,78,79,80,81,82,83,84,85,86,87,88,89,90,91,92,93,94,95,96,97,98,99,100,101,102,103,104,105,106,107,108,109,110,111,112,113,114].
