# Supplementary material for: Time-Restricted Eating and Bone Health: A Systematic Review with Meta-Analysis
Source: Nutrients. 2024 Mar 18;16(6):876. doi: 10.3390/nu16060876 (PMC10974430; doi:10.3390/nu16060876)

Time-restricted eating and bone health: A systematic review with meta-analysis

Rubén Fernández-Rodríguez [1], Miriam Garrido-Miguel [1,2], Bruno Bizzozero-Peroni [1,3*], Valentina Díaz-Goñi[1], Eva Rodríguez-Gutiérrez [1], María José Guzmán-Pavón [4], Ana Belén Meseguer-Henarejos [5], Ana Torres-Costoso[1,4]

1. Health and Social Research Center, Universidad de Castilla-La Mancha, 16002 Cuenca, Spain; Ru-ben.Fernandez@uclm.es

2. Research Network on Chronicity, Primary Care and Health Promotion (RICAPPS), Cuenca, Spain.

3. Faculty of Nursing, Universidad de Castilla-La Mancha, 02006, Albacete, Spain.

4. Instituto Superior de Educación Física, Universidad de la República, 40000, Rivera, Uruguay.

5. Faculty of Physiotherapy and Nursing, Universidad de Castilla-La Mancha, 45071, Toledo, Spain.

6. Faculty of Medicine, Department of Physiotherapy, Universidad de Murcia, 30120, Spain.

*Correspondence: Bruno.Bizzozero@uclm.es; Tel.: +34-969179100

Funding: RFR is supported by a grant from the Spanish Ministry of Education, Culture and Sport (FPU 19/00167). BBP is supported by a grant from the University of Castilla-La Mancha co-financed by the European Social Fund (2020-PREDUCLM-16746). ERG is supported by a grant from the University of Castilla-La Mancha (2022-UNIVERS-11373). VDG is supported by a grant from the National Agency for Research and Innovation of Uruguay (grant number POS_EXT_2023 _1_175630)

Index:

ID	SUBHEADING	
**PRISMA**	Prisma checklist.	3-5
**TABLE S1**	Search strategy for each database.	6
**TABLE S2**	Studies excluded after full-text reading with reasons.	7-15
**TABLE S3**	Effects of time-restricted eating interventions on bone mineral content compared to control conditions.	16
**TABLE S4**	Effects of time-restricted eating interventions on bone turnover markers compared to control conditions.	17
**TABLE S5**	Effects of time-restricted eating interventions on bone mineral density compared to control conditions.	18
**TABLE S6**	Effects of time-restricted eating interventions on fat mass compared to control conditions.	19
**TABLE S7**	Effects of time-restricted eating interventions on lean mass compared to control conditions.	20
**TABLE S8**	Sensitivity analyses using the leave-one-out method.	21
**TABLE S9**	Sensitivity analysis after maintaining the predominant health status and time-restricted eating protocol among the included studies.	22
**TABLE S10**	GRADE assessment.	23
**FIGURE S1**	Risk of bias assessment	24

Section and Topic	Item #	Checklist item	Location where item is reported
**TITLE**			
Title	1	Identify the report as a systematic review.	Page 1
**ABSTRACT**			
Abstract	2	See the PRISMA 2020 for Abstracts checklist.	1
**INTRODUCTION**			
Rationale	3	Describe the rationale for the review in the context of existing knowledge.	1-2
Objectives	4	Provide an explicit statement of the objective(s) or question(s) the review addresses.	2
**METHODS**			
Eligibility criteria	5	Specify the inclusion and exclusion criteria for the review and how studies were grouped for the syntheses.	3
Information sources	6	Specify all databases, registers, websites, organisations, reference lists and other sources searched or consulted to identify studies. Specify the date when each source was last searched or consulted.	2
Search strategy	7	Present the full search strategies for all databases, registers and websites, including any filters and limits used.	3<br>Table S1
Selection process	8	Specify the methods used to decide whether a study met the inclusion criteria of the review, including how many reviewers screened each record and each report retrieved, whether they worked independently, and if applicable, details of automation tools used in the process.	3
Data collection process	9	Specify the methods used to collect data from reports, including how many reviewers collected data from each report, whether they worked independently, any processes for obtaining or confirming data from study investigators, and if applicable, details of automation tools used in the process.	3
Data items	10a	List and define all outcomes for which data were sought. Specify whether all results that were compatible with each outcome domain in each study were sought (e.g. for all measures, time points, analyses), and if not, the methods used to decide which results to collect.	3
	10b	List and define all other variables for which data were sought (e.g. participant and intervention characteristics, funding sources). Describe any assumptions made about any missing or unclear information.	3
Study risk of bias assessment	11	Specify the methods used to assess risk of bias in the included studies, including details of the tool(s) used, how many reviewers assessed each study and whether they worked independently, and if applicable, details of automation tools used in the process.	3
Effect measures	12	Specify for each outcome the effect measure(s) (e.g. risk ratio, mean difference) used in the synthesis or presentation of results.	3-4
Synthesis methods	13a	Describe the processes used to decide which studies were eligible for each synthesis (e.g. tabulating the study intervention characteristics and comparing against the planned groups for each synthesis (item #5)).	4
	13b	Describe any methods required to prepare the data for presentation or synthesis, such as handling of missing summary statistics, or data conversions.	4

Section and Topic	Item #	Checklist item	Location where item is reported
	13c	Describe any methods used to tabulate or visually display results of individual studies and syntheses.	4
	13d	Describe any methods used to synthesize results and provide a rationale for the choice(s). If meta-analysis was performed, describe the model(s), method(s) to identify the presence and extent of statistical heterogeneity, and software package(s) used.	3-4
	13e	Describe any methods used to explore possible causes of heterogeneity among study results (e.g. subgroup analysis, meta-regression).	3
	13f	Describe any sensitivity analyses conducted to assess robustness of the synthesized results.	4
Reporting bias assessment	14	Describe any methods used to assess risk of bias due to missing results in a synthesis (arising from reporting biases).	4
Certainty assessment	15	Describe any methods used to assess certainty (or confidence) in the body of evidence for an outcome.	4
**RESULTS**			
Study selection	16a	Describe the results of the search and selection process, from the number of records identified in the search to the number of studies included in the review, ideally using a flow diagram.	4-5<br>Figure 1
	16b	Cite studies that might appear to meet the inclusion criteria, but which were excluded, and explain why they were excluded.	4<br>Table S2
Study characteristics	17	Cite each included study and present its characteristics.	5<br>Table 1
Risk of bias in studies	18	Present assessments of risk of bias for each included study.	10<br>Figure S1
Results of individual studies	19	For all outcomes, present, for each study: (a) summary statistics for each group (where appropriate) and (b) an effect estimate and its precision (e.g. confidence/credible interval), ideally using structured tables or plots.	9<br>Figures S3-S7
Results of syntheses	20a	For each synthesis, briefly summarise the characteristics and risk of bias among contributing studies.	NA
	20b	Present results of all statistical syntheses conducted. If meta-analysis was done, present for each the summary estimate and its precision (e.g. confidence/credible interval) and measures of statistical heterogeneity. If comparing groups, describe the direction of the effect.	10<br>Figure 2
	20c	Present results of all investigations of possible causes of heterogeneity among study results.	NA
	20d	Present results of all sensitivity analyses conducted to assess the robustness of the synthesized results.	10<br>Table S8
Reporting biases	21	Present assessments of risk of bias due to missing results (arising from reporting biases) for each synthesis assessed.	NA

Section and Topic	Item #	Checklist item	Location where item is reported
Certainty of evidence	22	Present assessments of certainty (or confidence) in the body of evidence for each outcome assessed.	10<br>Table S10
**DISCUSSION**			
Discussion	23a	Provide a general interpretation of the results in the context of other evidence.	10-11
	23b	Discuss any limitations of the evidence included in the review.	11
	23c	Discuss any limitations of the review processes used.	12
	23d	Discuss implications of the results for practice, policy, and future research.	12
**OTHER INFORMATION**			
Registration and protocol	24a	Provide registration information for the review, including register name and registration number, or state that the review was not registered.	2
	24b	Indicate where the review protocol can be accessed, or state that a protocol was not prepared.	NA
	24c	Describe and explain any amendments to information provided at registration or in the protocol.	NA
Support	25	Describe sources of financial or non-financial support for the review, and the role of the funders or sponsors in the review.	12
Competing interests	26	Declare any competing interests of review authors.	12
Availability of data, code and other materials	27	Report which of the following are publicly available and where they can be found: template data collection forms; data extracted from included studies; data used for all analyses; analytic code; any other materials used in the review.	12

Table S1. Search strategy for each database.

Date: October 1st, 2023.

MEDLINE (via Pubmed):

("time-restricted eating"[All Fields] OR "time-restricted feeding"[All Fields]) AND ("bone"[All Fields] OR "bone mineral density"[All Fields] OR ("osteoporosis"[MeSH Terms] OR "osteoporosis"[All Fields] OR "osteoporoses"[All Fields] OR "osteoporosis, postmenopausal"[MeSH Terms] OR ("osteoporosis"[All Fields] AND "postmenopausal"[All Fields]) OR "postmenopausal osteoporosis"[All Fields]) OR ("fractur"[All Fields] OR "fractural"[All Fields] OR "fracture s"[All Fields] OR "fractures, bone"[MeSH Terms] OR ("fractures"[All Fields] AND "bone"[All Fields]) OR "bone fractures"[All Fields] OR "fracture"[All Fields] OR "fractured"[All Fields] OR "fractures"[All Fields] OR "fracturing"[All Fields]) OR "bone turnover"[All Fields] OR "bone remodelling"[All Fields]). **Results**= 11

("Time-restricted eating" OR "time-restricted feeding" OR "time limited eating") NOT review. Filter: Human. **Results**= 237

Cochrane:

Title abs key: ("Time-restricted eating" OR "time-restricted feeding" OR "time limited eating") AND ("bone" OR "bone turnover" OR "bone mineral density" OR "osteoporosis"). **Results**: 15

Scopus:

Title abs key: ("Time-restricted eating" OR "time-restricted feeding" OR "time limited eating") AND ("bone" OR "bone turnover" OR "bone mineral density" OR "osteoporosis"). **Results**: 16 (limited to article)*

WoS:

Topic: ("Time-restricted eating" OR "time-restricted feeding" OR "time limited eating") AND ("bone" OR "bone turnover" OR "bone mineral density" OR "osteoporosis"). **Results**: 9 (limited to article)

TOTAL RESULTS: 51+237=288

After duplicated removed with Endnote tool: 264 (removed = 24)

Excluded after Title-abstract screening: 196

Assessed full text: 68 (excluded 61)

Finally included in the review: 7

Table S2. Reason for exclusion after full-text screening (n= 61).

ID	Reference	Reason for exclusion
1	Allison, K. C., Hopkins, C. M., Ruggieri, M., Spaeth, A. M., Ahima, R. S., Zhang, Z., Taylor, D. M., & Goel, N. (2021). Prolonged, Controlled Daytime versus Delayed Eating Impacts Weight and Metabolism. Curr Biol, 31(3), 650-657.e653. https://doi.org/10.1016/j.cub.2020.10.092	Non-outcome of interest
2	Andriessen, C., Fealy, C. E., Veelen, A., van Beek, S. M. M., Roumans, K. H. M., Connell, N. J., Mevenkamp, J., Moonen-Kornips, E., Havekes, B., Schrauwen-Hinderling, V. B., Hoeks, J., & Schrauwen, P. (2022). Three weeks of time-restricted eating improves glucose homeostasis in adults with type 2 diabetes but does not improve insulin sensitivity: a randomised crossover trial. Diabetologia, 65(10), 1710-1720. https://doi.org/10.1007/s00125-022-05752-z	Non-outcome of interest
3	Bantle, A. E., Lau, K. J., Wang, Q., Malaeb, S., Harindhanavudhi, T., Manoogian, E. N. C., Panda, S., Mashek, D. G., & Chow, L. S. (2023). Time-restricted eating did not alter insulin sensitivity or β-cell function in adults with obesity: A randomized pilot study. Obesity (Silver Spring), 31 Suppl 1(Suppl 1), 108-115. https://doi.org/10.1002/oby.23620	Non-outcome of interest
4	Bao, R., Sun, Y., Jiang, Y., Ye, L., Hong, J., & Wang, W. (2022). Effects of Time-Restricted Feeding on Energy Balance: A Cross-Over Trial in Healthy Subjects. Front Endocrinol (Lausanne), 13, 870054. https://doi.org/10.3389/fendo.2022.870054	Non-outcome of interest
5	Brady, A. J., Langton, H. M., Mulligan, M., & Egan, B. (2021). Effects of 8 wk of 16:8 Time-restricted Eating in Male Middle- and Long-Distance Runners. Med Sci Sports Exerc, 53(3), 633-642. https://doi.org/10.1249/mss.0000000000002488	Non-outcome of interest
6	Chow, L. S., Manoogian, E. N. C., Alvear, A., Fleischer, J. G., Thor, H., Dietsche, K., Wang, Q., Hodges, J. S., Esch, N., Malaeb, S., Harindhanavudhi, T., Nair, K. S., Panda, S., & Mashek, D. G. (2020). Time-Restricted Eating Effects on Body Composition and Metabolic Measures in Humans who are Overweight: A Feasibility Study. Obesity (Silver Spring), 28(5), 860-869. https://doi.org/10.1002/oby.22756	Non-outcome of interest
7	Cienfuegos, S., Gabel, K., Kalam, F., Ezpeleta, M., Lin, S., & Varady, K. A. (2021). Changes in body weight and metabolic risk during time restricted	Non-outcome of interest

	feeding in premenopausal versus postmenopausal women. Exp Gerontol, 154, 111545. https://doi.org/10.1016/j.exger.2021.111545	
8	Cienfuegos, S., Gabel, K., Kalam, F., Ezpeleta, M., Pavlou, V., Lin, S., Wiseman, E., & Varady, K. A. (2022). The effect of 4-h versus 6-h time restricted feeding on sleep quality, duration, insomnia severity and obstructive sleep apnea in adults with obesity. Nutr Health, 28(1), 5-11. https://doi.org/10.1177/02601060211002347	Non-outcome of interest
9	Cienfuegos, S., Gabel, K., Kalam, F., Ezpeleta, M., Wiseman, E., Pavlou, V., Lin, S., Oliveira, M. L., & Varady, K. A. (2020). Effects of 4- and 6-h Time-Restricted Feeding on Weight and Cardiometabolic Health: A Randomized Controlled Trial in Adults with Obesity. Cell Metab, 32(3), 366-378.e363. https://doi.org/10.1016/j.cmet.2020.06.018	Non-outcome of interest
10	Correia, J. M., Santos, I., Pezarat-Correia, P., Minderico, C., Schoenfeld, B. J., & Mendonca, G. V. (2021). Effects of Time-Restricted Feeding on Supramaximal Exercise Performance and Body Composition: A Randomized and Counterbalanced Crossover Study in Healthy Men. Int J Environ Res Public Health, 18(14). https://doi.org/10.3390/ijerph18147227	Non-outcome of interest
11	Correia, J. M., Santos, P. D. G., Pezarat-Correia, P., Minderico, C. S., Infante, J., & Mendonca, G. V. (2023). Effect of Time-Restricted Eating and Resistance Training on High-Speed Strength and Body Composition. Nutrients, 15(2). https://doi.org/10.3390/nu15020285	Non-outcome of interest
12	Crose, A., Alvear, A., Singroy, S., Wang, Q., Manoogian, E., Panda, S., Mashek, D. G., & Chow, L. S. (2021). Time-Restricted Eating Improves Quality of Life Measures in Overweight Humans. Nutrients, 13(5). https://doi.org/10.3390/nu13051430	Non-outcome of interest
13	Da Silva, B. R., Kirkham, A. A., Ford, K. L., Haykowsky, M. J., Paterson, D. I., Joy, A. A., Pituskin, E., Thompson, R., & Prado, C. M. (2023). Time-Restricted Eating in Breast Cancer Survivors: Effects on Body Composition and Nutritional Status. Nutr Cancer, 75(5), 1309-1314. https://doi.org/10.1080/01635581.2023.2195543	Study design
14	de Oliveira Maranhão Pureza, I. R., da Silva Junior, A. E., Silva Praxedes, D. R., Lessa Vasconcelos, L. G., de Lima Macena, M., Vieira de Melo, I. S., de Menezes Toledo Florêncio, T. M., & Bueno, N. B. (2021). Effects of time-restricted feeding on body weight, body composition and vital signs in low-	Non-outcome of interest

	income women with obesity: A 12-month randomized clinical trial. Clin Nutr, 40(3), 759-766. https://doi.org/10.1016/j.clnu.2020.06.036	
15	Domaszewski, P., Konieczny, M., Dybek, T., Łukaniszyn-Domaszewska, K., Anton, S., Sadowska-Krępa, E., & Skorupska, E. (2023). Comparison of the effects of six-week time-restricted eating on weight loss, body composition, and visceral fat in overweight older men and women. Exp Gerontol, 174, 112116. https://doi.org/10.1016/j.exger.2023.112116	Non-outcome of interest
16	Domaszewski, P., Konieczny, M., Pakosz, P., Łukaniszyn-Domaszewska, K., Mikuláková, W., Sadowska-Krępa, E., & Anton, S. (2022). Effect of a six-week times restricted eating intervention on the body composition in early elderly men with overweight. Sci Rep, 12(1), 9816. https://doi.org/10.1038/s41598-022-13904-9	Non-outcome of interest
17	Ferrocino, I., Pellegrini, M., D'Eusebio, C., Goitre, I., Ponzo, V., Fadda, M., Rosato, R., Mengozzi, G., Beccuti, G., Merlo, F. D., Rahimi, F., Comazzi, I., Cocolin, L., Ghigo, E., & Bo, S. (2022). The Effects of Time-Restricted Eating on Metabolism and Gut Microbiota: A Real-Life Study. Nutrients, 14(13). https://doi.org/10.3390/nu14132569	Study design
18	Gasmi, M., Sellami, M., Denham, J., Padulo, J., Kuvacic, G., Selmi, W., & Khalifa, R. (2018). Time-restricted feeding influences immune responses without compromising muscle performance in older men. Nutrition, 51-52, 29-37. https://doi.org/10.1016/j.nut.2017.12.014	Non-outcome of interest
19	Gonzalez, A. E., Waldman, H. S., Abel, M. G., McCurdy, K. W., & McAllister, M. J. (2021). Impact of Time Restricted Feeding on Fitness Variables in Professional Resistance Trained Firefighters. J Occup Environ Med, 63(4), 343-349. https://doi.org/10.1097/jom.0000000000002144	Study design
20	Haganes, K. L., Silva, C. P., Eyjólfsdóttir, S. K., Steen, S., Grindberg, M., Lydersen, S., Hawley, J. A., & Moholdt, T. (2022). Time-restricted eating and exercise training improve HbA1c and body composition in women with overweight/obesity: A randomized controlled trial. Cell Metab, 34(10), 1457-1471.e1454. https://doi.org/10.1016/j.cmet.2022.09.003	Non-outcome of interest
21	He, M., Wang, J., Liang, Q., Li, M., Guo, H., Wang, Y., Deji, C., Sui, J., Wang, Y. W., Liu, Y., Zheng, Y., Qian, B., Chen, H., Ma, M., Su, S., Geng, H., Zhou, W. X., Guo, X., Zhu, W. Z., . . . Shi, B. (2022). Time-restricted eating with or without low-carbohydrate diet reduces visceral fat and improves metabolic	Non-outcome of interest

	syndrome: A randomized trial. Cell Rep Med, 3(10), 100777. https://doi.org/10.1016/j.xcrm.2022.100777	
22	Hutchison, A. T., Regmi, P., Manoogian, E. N. C., Fleischer, J. G., Wittert, G. A., Panda, S., & Heilbronn, L. K. (2019). Time-Restricted Feeding Improves Glucose Tolerance in Men at Risk for Type 2 Diabetes: A Randomized Crossover Trial. Obesity (Silver Spring), 27(5), 724-732. https://doi.org/10.1002/oby.22449	Study design
23	Isenmann, E., Dissemond, J., & Geisler, S. (2021). The Effects of a Macronutrient-Based Diet and Time-Restricted Feeding (16:8) on Body Composition in Physically Active Individuals-A 14-Week Randomised Controlled Trial. Nutrients, 13(9). https://doi.org/10.3390/nu13093122	Non-outcome of interest
24	Jamshed, H., Steger, F. L., Bryan, D. R., Richman, J. S., Warriner, A. H., Hanick, C. J., Martin, C. K., Salvy, S. J., & Peterson, C. M. (2022). Effectiveness of Early Time-Restricted Eating for Weight Loss, Fat Loss, and Cardiometabolic Health in Adults With Obesity: A Randomized Clinical Trial. JAMA Intern Med, 182(9), 953-962. https://doi.org/10.1001/jamainternmed.2022.3050	Non-outcome of interest
25	Kesztyüs, D., Cermak, P., Gulich, M., & Kesztyüs, T. (2019). Adherence to Time-Restricted Feeding and Impact on Abdominal Obesity in Primary Care Patients: Results of a Pilot Study in a Pre-Post Design. Nutrients, 11(12). https://doi.org/10.3390/nu11122854	Study design
26	Li, C., Xing, C., Zhang, J., Zhao, H., Shi, W., & He, B. (2021). Eight-hour time-restricted feeding improves endocrine and metabolic profiles in women with anovulatory polycystic ovary syndrome. J Transl Med, 19(1), 148. https://doi.org/10.1186/s12967-021-02817-2	Study design
27	Lin, S., Cienfuegos, S., Ezpeleta, M., Gabel, K., Pavlou, V., Mulas, A., Chakos, K., McStay, M., Wu, J., Tussing-Humphreys, L., Alexandria, S. J., Sanchez, J., Unterman, T., & Varady, K. A. (2023). Time-Restricted Eating Without Calorie Counting for Weight Loss in a Racially Diverse Population : A Randomized Controlled Trial. Ann Intern Med, 176(7), 885-895. https://doi.org/10.7326/m23-0052	Non-outcome of interest
28	Lin, Y. J., Wang, Y. T., Chan, L. C., & Chu, N. F. (2022). Effect of time-restricted feeding on body composition and cardio-metabolic risk in middle-aged women in Taiwan. Nutrition, 93, 111504. https://doi.org/10.1016/j.nut.2021.111504	Non-outcome of interest

29	Liu, D., Huang, Y., Huang, C., Yang, S., Wei, X., Zhang, P., Guo, D., Lin, J., Xu, B., Li, C., He, H., He, J., Liu, S., Shi, L., Xue, Y., & Zhang, H. (2022). Calorie Restriction with or without Time-Restricted Eating in Weight Loss. N Engl J Med, 386(16), 1495-1504. https://doi.org/10.1056/NEJMoa2114833	Non-outcome of interest
30	Liu, H. T., Chen, S. Y., Ji, H. Y., & Dai, Z. Q. (2023). Effects of time-restricted feeding and walking exercise on the physical health of female college students with hidden obesity: a randomized trial. Frontiers in Public Health, 11, Article 1020887. https://doi.org/10.3389/fpubh.2023.1020887	Duplicated
31	Lobene, A., Panda, S., Mashek, D., Gallant, K. H., & Chow, L. (2020). Time Restricted Eating for 12 Weeks Does Not Adversely Alter Bone Mineral Content and Bone Metabolism in Overweight Adults [Journal article; Conference proceeding]. Journal of bone and mineral research, 35(SUPPL 1), 232-233. https://doi.org/10.1002/jbmr.4206	Duplicated
32	Lobene, A. J., Panda, S., Mashek, D. G., Manoogian, E. N. C., Hill Gallant, K. M., & Chow, L. S. (2021). Time-Restricted Eating for 12 Weeks Does Not Adversely Alter Bone Turnover in Overweight Adults. Nutrients, 13(4). https://doi.org/10.3390/nu13041155	Duplicated
33	Manoogian, E. N. C., Zadourian, A., Lo, H. C., Gutierrez, N. R., Shoghi, A., Rosander, A., Pazargadi, A., Ormiston, C. K., Wang, X., Sui, J., Hou, Z., Fleischer, J. G., Golshan, S., Taub, P. R., & Panda, S. (2022). Feasibility of time-restricted eating and impacts on cardiometabolic health in 24-h shift workers: The Healthy Heroes randomized control trial. Cell Metab, 34(10), 1442-1456.e1447. https://doi.org/10.1016/j.cmet.2022.08.018	Non-outcome of interest
34	Martens, C. R., Rossman, M. J., Mazzo, M. R., Jankowski, L. R., Nagy, E. E., Denman, B. A., Richey, J. J., Johnson, S. A., Ziemba, B. P., Wang, Y., Peterson, C. M., Chonchol, M., & Seals, D. R. (2020). Short-term time-restricted feeding is safe and feasible in non-obese healthy midlife and older adults. Geroscience, 42(2), 667-686. https://doi.org/10.1007/s11357-020-00156-6	Duplicated
35	Mayra, S. T., Chondropoulos, K., De Leon, A., Kravat, N., & Johnston, C. S. (2022). The feasibility and preliminary efficacy of early time-restricted eating on diet quality in college students: A randomized study. Obes Res Clin Pract, 16(5), 413-420. https://doi.org/10.1016/j.orcp.2022.08.009	Non-outcome of interest
36	McAllister, M. J., Pigg, B. L., Renteria, L. I., & Waldman, H. S. (2020). Time-restricted feeding improves markers of cardiometabolic health in physically	Study design

	active college-age men: a 4-week randomized pre-post pilot study. Nutr Res, 75, 32-43. https://doi.org/10.1016/j.nutres.2019.12.001	
37	Moro, T., Tinsley, G., Bianco, A., Marcolin, G., Pacelli, Q. F., Battaglia, G., Palma, A., Gentil, P., Neri, M., & Paoli, A. (2016). Effects of eight weeks of time-restricted feeding (16/8) on basal metabolism, maximal strength, body composition, inflammation, and cardiovascular risk factors in resistance-trained males. J Transl Med, 14(1), 290. https://doi.org/10.1186/s12967-016-1044-0	Non-outcome of interest
38	Moro, T., Tinsley, G., Longo, G., Grigoletto, D., Bianco, A., Ferraris, C., Guglielmetti, M., Veneto, A., Tagliabue, A., Marcolin, G., & Paoli, A. (2020). Time-restricted eating effects on performance, immune function, and body composition in elite cyclists: a randomized controlled trial. J Int Soc Sports Nutr, 17(1), 65. https://doi.org/10.1186/s12970-020-00396-z	Non-outcome of interest
39	Moro, T., Tinsley, G., Pacelli, F. Q., Marcolin, G., Bianco, A., & Paoli, A. (2021). Twelve Months of Time-restricted Eating and Resistance Training Improves Inflammatory Markers and Cardiometabolic Risk Factors. Med Sci Sports Exerc, 53(12), 2577-2585. https://doi.org/10.1249/mss.0000000000002738	Non-outcome of interest
40	Papageorgiou, M., Biver, E., Mareschal, J., Phillips, N. E., Hemmer, A., Biolley, E., Schwab, N., Manoogian, E. N. C., Gonzalez Rodriguez, E., Aeberli, D., & et al. (2023). The Effects Of Time-Restricted Eating (Tre) And Weight Loss On Bone Metabolism And Health: an Exploratory Analysis In A 6-Month Randomised Controlled Trial [Journal article; Conference proceeding]. Clinical nutrition ESPEN, 54, 558. https://doi.org/10.1016/j.clnesp.2022.09.296	Duplicated
41	Papageorgiou, M., Biver, E., Mareschal, J., Phillips, N. E., Hemmer, A., Biolley, E., Schwab, N., Manoogian, E. N. C., Gonzalez Rodriguez, E., Aeberli, D., & et al. (2023). The effects of time-restricted eating and weight loss on bone metabolism and health: a 6-month randomized controlled trial [Journal article]. Obesity (Silver Spring, Md.), 31 Suppl 1(Suppl 1), 85-95. https://doi.org/10.1002/oby.23577	Duplicated
42	Papageorgiou, M., Biver, E., Mareschal, J., Phillips, N. E., Hemmer, A., Biolley, E., Schwab, N., Manoogian, E. N. C., Rodriguez, E. G., Aeberli, D., Hans, D., Pot, C., Panda, S., Rodondi, N., Ferrari, S. L., & Collet, T. H. (2023). The effects of time-restricted eating and weight loss on bone metabolism and	Duplicated

	health: a 6-month randomized controlled trial. OBESITY, 31, 85-95. https://doi.org/10.1002/oby.23577	
43	Parr, E., Kouw, I., Wheeler, M., Radford, B., Hall, R., Senden, J., Goessens, J., Van Loon, L., & Hawley, J. (2022). Short-term time-restricted eating does not lower muscle protein synthesis rates in men with overweight/obesity [Journal article; Conference proceeding]. Obesity reviews, 23. https://doi.org/10.1111/obr.13502	Study design
44	Phillips, N. E., Mareschal, J., Schwab, N., Manoogian, E. N. C., Borloz, S., Ostinelli, G., Gauthier-Jaques, A., Umwali, S., Gonzalez Rodriguez, E., Aeberli, D., Hans, D., Panda, S., Rodondi, N., Naef, F., & Collet, T. H. (2021). The Effects of Time-Restricted Eating versus Standard Dietary Advice on Weight, Metabolic Health and the Consumption of Processed Food: A Pragmatic Randomised Controlled Trial in Community-Based Adults. Nutrients, 13(3). https://doi.org/10.3390/nu13031042	Non-outcome of interest
45	Pureza, I., Melo, I. S. V., Macena, M. L., Praxedes, D. R. S., Vasconcelos, L. G. L., Silva-Júnior, A. E., Florêncio, T., & Bueno, N. B. (2020). Acute effects of time-restricted feeding in low-income women with obesity placed on hypoenergetic diets: Randomized trial. Nutrition, 77, 110796. https://doi.org/10.1016/j.nut.2020.110796	Non-intervention of interest
46	Steger, F. L., Jamshed, H., Bryan, D. R., Richman, J. S., Warriner, A. H., Hanick, C. J., Martin, C. K., Salvy, S. J., & Peterson, C. M. (2023). Early time-restricted eating affects weight, metabolic health, mood, and sleep in adherent completers: A secondary analysis. Obesity (Silver Spring), 31 Suppl 1(Suppl 1), 96-107. https://doi.org/10.1002/oby.23614	Duplicated
47	Steger, F. L., Jamshed, H., Martin, C. K., Richman, J. S., Bryan, D. R., Hanick, C. J., Salvy, S. J., Warriner, A. H., & Peterson, C. M. (2023). Impact of early time-restricted eating on diet quality, meal frequency, appetite, and eating behaviors: A randomized trial. Obesity (Silver Spring), 31 Suppl 1(Suppl 1), 127-138. https://doi.org/10.1002/oby.23642	Non-outcome of interest
48	Stratton, M. T., Tinsley, G. M., Alesi, M. G., Hester, G. M., Olmos, A. A., Serafini, P. R., Modjeski, A. S., Mangine, G. T., King, K., Savage, S. N., & et al. (2020). Four weeks of time-restricted feeding combined with resistance training does not differentially influence measures of body composition, muscle performance, resting energy expenditure, and blood biomarkers [Journal article]. Nutrients, 12(4). https://doi.org/10.3390/nu12041126	Study design

49	Stratton, M. T., Tinsley, G. M., Alesi, M. G., Hester, G. M., Olmos, A. A., Serafini, P. R., Modjeski, A. S., Mangine, G. T., King, K., Savage, S. N., Webb, A. T., & VanDusseldorp, T. A. (2020). Four Weeks of Time-Restricted Feeding Combined with Resistance Training Does Not Differentially Influence Measures of Body Composition, Muscle Performance, Resting Energy Expenditure, and Blood Biomarkers. Nutrients, 12(4). https://doi.org/10.3390/nu12041126	Duplicated
50	Sutton, E. F., Beyl, R., Early, K. S., Cefalu, W. T., Ravussin, E., & Peterson, C. M. (2018). Early Time-Restricted Feeding Improves Insulin Sensitivity, Blood Pressure, and Oxidative Stress Even without Weight Loss in Men with Prediabetes. Cell Metab, 27(6), 1212-1221.e1213. https://doi.org/10.1016/j.cmet.2018.04.010	Non-outcome of interest
51	Teong, X. T., Liu, K., Vincent, A. D., Bensalem, J., Liu, B., Hattersley, K. J., Zhao, L., Feinle-Bisset, C., Sargeant, T. J., Wittert, G. A., Hutchison, A. T., & Heilbronn, L. K. (2023). Intermittent fasting plus early time-restricted eating versus calorie restriction and standard care in adults at risk of type 2 diabetes: a randomized controlled trial. Nat Med, 29(4), 963-972. https://doi.org/10.1038/s41591-023-02287-7	Non-outcome of interest
52	Thomas, E. A., Zaman, A., Sloggett, K. J., Steinke, S., Grau, L., Catenacci, V. A., Cornier, M. A., & Rynders, C. A. (2022). Early time-restricted eating compared with daily caloric restriction: A randomized trial in adults with obesity. Obesity (Silver Spring), 30(5), 1027-1038. https://doi.org/10.1002/oby.23420	Non-outcome of interest
53	Tinsley, G. M., Forsse, J. S., Butler, N. K., Paoli, A., Bane, A. A., La Bounty, P. M., Morgan, G. B., & Grandjean, P. W. (2017). Time-restricted feeding in young men performing resistance training: A randomized controlled trial. Eur J Sport Sci, 17(2), 200-207. https://doi.org/10.1080/17461391.2016.1223173	Non-outcome of interest
54	Tinsley, G. M., Moore, M. L., Graybeal, A. J., Paoli, A., Kim, Y., Gonzales, J. U., Harry, J. R., VanDusseldorp, T. A., Kennedy, D. N., & Cruz, M. R. (2019). Time-restricted feeding plus resistance training in active females: a randomized trial. Am J Clin Nutr, 110(3), 628-640. https://doi.org/10.1093/ajcn/nqz126	Non-outcome of interest
55	Tinsley, G. M., & Paoli, A. (2019). Time-restricted eating and age-related muscle loss. Aging (Albany NY), 11(20), 8741-8742. https://doi.org/10.18632/aging.102384	Study design

56	Tovar, A. P., Richardson, C. E., Keim, N. L., Van Loan, M. D., Davis, B. A., & Casazza, G. A. (2021). Four Weeks of 16/8 Time Restrictive Feeding in Endurance Trained Male Runners Decreases Fat Mass, without Affecting Exercise Performance. Nutrients, 13(9). https://doi.org/10.3390/nu13092941	Non-outcome of interest
57	Valenzuela, P. L., Castillo-García, A., & Lucia, A. (2022). Calorie Restriction with or without Time-Restricted Eating in Weight Loss. N Engl J Med, 387(3), 280-281. https://doi.org/10.1056/NEJMc2207023	Study design
58	Vidmar, A. P., Naguib, M., Raymond, J. K., Salvy, S. J., Hegedus, E., Wee, C. P., & Goran, M. I. (2021). Time-Limited Eating and Continuous Glucose Monitoring in Adolescents with Obesity: A Pilot Study. Nutrients, 13(11). https://doi.org/10.3390/nu13113697	Non-outcome of interest
59	Wei, X., Lin, B., Huang, Y., Yang, S., Huang, C., Shi, L., Liu, D., Zhang, P., Lin, J., Xu, B., Guo, D., Li, C., He, H., Liu, S., Xue, Y., Xu, Y., & Zhang, H. (2023). Effects of Time-Restricted Eating on Nonalcoholic Fatty Liver Disease: The TREATY-FLD Randomized Clinical Trial. JAMA Netw Open, 6(3), e233513. https://doi.org/10.1001/jamanetworkopen.2023.3513	Non-outcome of interest
60	Xie, Z., Sun, Y., Ye, Y., Hu, D., Zhang, H., He, Z., Zhao, H., Yang, H., & Mao, Y. (2022). Randomized controlled trial for time-restricted eating in healthy volunteers without obesity. Nat Commun, 13(1), 1003. https://doi.org/10.1038/s41467-022-28662-5	Non-outcome of interest
61	Zeb, F., Wu, X., Chen, L., Fatima, S., Ijaz Ul, H., Chen, A., Xu, C., Jianglei, R., Feng, Q., & Li, M. (2020). Time-restricted feeding is associated with changes in human gut microbiota related to nutrient intake. Nutrition, 78, 110797. https://doi.org/10.1016/j.nut.2020.110797	Non-outcome of interest

Table S3. Effects of time-restricted eating interventions on bone mineral content compared to control conditions.

						Mean (SE) [1]	
**Study**	**Population**	**TRE**	**CON**	**n** $_{TRE/CON}$	**Length** wk	**TRE** $_{pre/post}$	**CON** $_{pre/post}$
Kotarsky et al. (2021) [42]	Ov-Ob	16:8	Non-TRE	11/10	8	2600 (110) / 2580 (110)	2580 (130) / 2570 (120)
Lobene et al. (2021) [44]	Ov-Ob	16:8	Non-TRE	11/9	12	2852 (173) / 2866 (162)	2801 (159) / 2784 (159)
Lowe et al. (2023) [47]	Ov-Ob	16:8	Non-TRE	24/22	12	2511 (78) / 2523 (78)	2542 (78) / 2547 (78)
Papageorgiou et al. (2023) [45]	≥1 component of MetS	12:12	Non-TRE	23/19	24	2595 (97) / 2591 (94)	2548 (118) / 2543 (108)

[1] Pre-post means (standard errors) of total body bone mineral content (g) in TRE and non-TRE groups.

Abbreviations: CON, control group; **MetS**, metabolic syndrome; **Ob**, obesity; **Ov**, overweight; **SE**, standard error; **TRE,** time-restricted eating; **wk,** weeks.

Table S4. Effects of time-restricted eating interventions on bone turnover markers compared to control conditions.

			**Mean (SE)** [1]	
**Study**	**Bone turnover marker**	**Measure**	**TRE** $_{pre/post}$	**CON** $_{pre/post}$
Papageorgiou et al. (2023) [45]	CTX	ng/L	397 (26) / 388 (31)	407 (33) / 431.3 (44)
Lobene et al. (2021) [44]	P1NP	ng/mL	18.7 (1.3) / 18.5 (1.5)	28.8 (8.8) / 24.7 (8.2)
Papageorgiou et al. (2023) [45]		ug/L	54.4 (4.7) / 51.4 (4.3)	51.4 (2.9) / 48.5 (4.0)
Lobene et al. (2021) [44]	NTX	pg/mL	1074 (112) / 1102 (104)	1780 (513) / 1722 (327)

[1] Pre-post means (standard errors) of bone turnover markers in TRE and non-TRE groups.

Abbreviations: CON, control group; **CTX,** C-terminal telopeptide of type I collagen; **NTX,** N-terminal telopeptide of type I collagen; **P1NP,** N-terminal propeptide of type I procollagen; **SE**, standard error; **TRE,** time-restricted eating; **wk,** weeks.

Table S5. Effects of time-restricted eating interventions on bone mineral density compared to control conditions.

Study	Population	TRE	CON	n TRE/CON	Length wk	MD (SD) [1] TRE	MD (SD) [1] CON
Kotarsky et al. (2021) [42]	Ov-Ob	16:8	Non-TRE	11/10	8	-0.010 (0.060)	0.010 (0.016)
Liu et al. (2023) [43]	Ob	16:8	Non-TRE	19/19	8	0.000 (0.087)	0.010 (0.087)
Liu et al. (2023b) [43]	Ob	16:8	Non-TRE + Ex	19/20	8	0.000 (0.087)	0.010 (0.089)
Lobene et al. (2021) [44]	Ov-Ob	16:8	Non-TRE	11/9	12	0.002 (0.033)	0.001 (0.150)
Papageorgiou et al. (2023) [45]	≥1 component of MetS	12:12	Non-TRE	18/15	24	0.000 (0.063)	0.010 (0.027)
Richardson et al. (2023) [46]	Endurance-trained runners	16:8	Non-TRE	15/15	4	0.001 (0.046)	-0.001 (0.046)

[1] Pre-post mean differences (standard deviations) of total body bone mineral density (g/cm^2) in TRE and non-TRE groups.

Abbreviations: CON, control group; **Ex**, exercise; **MD**, mean difference; **MetS**, metabolic syndrome; **Ob**, obesity; **Ov**, overweight; **SD**, standard deviation; **TRE,** time-restricted eating; **wk,** weeks.

Table S6. Effects of time-restricted eating interventions on fat mass compared to control conditions.

						MD (SD) [1]		
**Study**	**Population**	**TRE**	**CON**	**n $_{TRE/CON}$**	**Length $_{wk}$**	**TRE**	**CON**	**MD (95% CI)**
Kotarsky et al. (2021) [42]	Ov-Ob	16:8	Non-TRE	11/10	8	-3.0 (9.4)	-1.0 (8.9)	-2.0 (-9.8 to 5.8)
Liu et al. (2023) [43]	Ob	16:8	Non-TRE	19/19	8	-0.3 (40.3)	0.4 (59.6)	-0.6 (-33.0 to 31.7)
Liu et al. (2023b) [43]	Ob	16:8	Non-TRE + Ex	19/20	8	-0.2 (38.2)	0.3 (41.5)	-0.5 (-25.5 to 24.5)
Lobene et al. (2021) [44]	Ov-Ob	16:8	Non-TRE	11/9	12	-1.7 (23.5)	-0.9 (29.3)	-0.8 (-24.5 to 22.9)
Lowe et al. (2023) [47]	Ov-Ob	16:8	Non-TRE	24/22	12	-0.5 (1.6)	-0.03 (1.5)	-0.5 (-1.4 to 0.4)
Richardson et al. (2023) [46]	Endurance-trained runners	16:8	Non-TRE	15/15	4	-0.8 (1.3)	0.1 (4.3)	-0.9 (-3.2 to 1.4)
**Random effects model I^2 = 0%**	-	16:8	Non-TRE	99/95	4 to 12	-	-	**-0.56 (-1.40 to 0.29)**

[1] Pre-post mean differences (standard deviations) of fat mass (kg) in TRE and non-TRE groups.

Abbreviations: CON, control group; **Ex**, exercise; **MetS**, metabolic syndrome; **Ob**, obesity; **Ov**, overweight; **SE**, standard error; **TRE,** time-restricted eating; **wk,** weeks.

Table S7. Effects of time-restricted eating interventions on lean mass compared to control conditions.

Study	Population	TRE	CON	n TRE/CON	Length wk	MD (SD) [1] TRE	MD (SD) [1] CON	MD (95% CI)
Kotarsky et al. (2021) [42]	Ov-Ob	16:8	Non-TRE	11/10	8	0.0 (9.4)	1.0 (8.9)	-1.0 (-8.8 to 6.8)
Liu et al. (2023) [43]	Ob	16:8	Non-TRE	19/19	8	-2.8 (39.0)	-1.1 (36.1)	-1.6 (-25.5 to 22.2)
Liu et al. (2023b) [43]	Ob	16:8	Non-TRE + Ex	19/20	8	-2.3 (35.8)	-2.3 (43.6)	-0.1 (-25.0 to 24.9)
Lobene et al. (2021) [44]	Ov-Ob	16:8	Non-TRE	11/9	12	-1.4 (13.9)	-0.1 (11.7)	-1.3 (-12.5 to 9.9)
Lowe et al. (2023) [47]	Ov-Ob	16:8	Non-TRE	24/22	12	-1.1 (1.6)	-0.4 (1.4)	-0.7 (-1.6 to 0.1)
**Random effects model I^2 = 0%**	Ov-Ob	16:8	Non-TRE	84/84	8 to 12	-	-	**-0.76 (-1.61 to 0.10)**

[1] Pre-post mean differences (standard deviations) of lean mass (kg) in TRE and non-TRE groups.

Abbreviations: CON, control group; **Ex**, exercise; **MetS**, metabolic syndrome; **Ob**, obesity; **Ov**, overweight; **SE**, standard error; **TRE,** time-restricted eating; **wk,** weeks.

Table S8. Sensitivity analyses using the leave-one-out method.

**Reference**	**MD** [1]	**LL**	**UL**	***p***	**I^2 (%)**
All studies	-0.009	-0.026	0.009	0.328	0
Omitting:					
Kotarsky et al. (2021) [42]	-0.005	-0.025	0.014	0.587	0
Liu et al. (2023) [43]	-0.008	-0.027	0.010	0.361	0
Liu et al. (2023b) [43]	-0.008	-0.027	0.010	0.361	0
Lobene et al. (2021) [44]	-0.009	-0.026	0.009	0.319	0
Papageorgiou et al. (2023) [45]	-0.008	-0.028	0.012	0.441	0
Richardson et al. (2023) [46]	-0.013	-0.033	0.008	0.223	0

[1] Mean difference in bone mineral density (g/cm^2) after comparing TRE and non-TRE groups.

Abbreviations: LL, lower limit; **MD**, mean difference; **UL**, upper limit.

Table S9. Sensitivity analysis after maintaining the predominant health status and time-restricted eating protocol among the included studies.

Sensitivity analysis	Data	n	MD (95% CI) [1]	*p*	I^2 (%)
Predominant health status	Ov-Ob	5	-0.013 (-0.033 to 0.008)	0.223	0
Predominant TRE protocol	16:8	5	-0.008 (-0.028 to 0.012)	0.441	0

[1] Mean difference in bone mineral density (g/cm^2) after comparing TRE and non-TRE groups.

n represents the number of comparisons included in each sensitivity analysis.

Abbreviations: CI, confidence interval; **MD**, mean difference; **Ob**, obesity; **Ov**, Overweight; **TRE**, time-restricted eating.

Table S10. Quality of evidence assessment according to the GRADE approach.

Certainty assessment							№ of patients		Effect (95% CI)	Certainty	Importance of the outcome
Outcome	№ of studies	Risk of bias	Heterogeneity and inconsistency	Indirectness	Imprecision	Publication bias	Intervention	Control			
Bone mineral density	6	Serious. All studies rated as "some concerns"	Not serious. $I^2 = 0.0\%$	Not serious [a]	Serious [b]	Not available	93	88	-0.009 (-0.026, 0.009)	⊕⊕◯◯ Low (downgrade by 2 levels for risk of bias and imprecision)	Important

Abbreviations: CI: confidence interval.

[a] Population, intervention, and outcomes did not differ across studies.
[b] The confidence interval of the effect size contained a null value.

Figure S1. Risk of bias assessment.

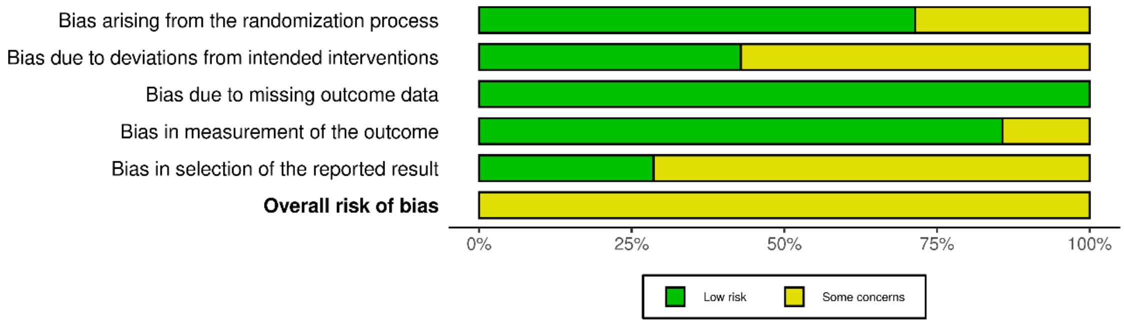

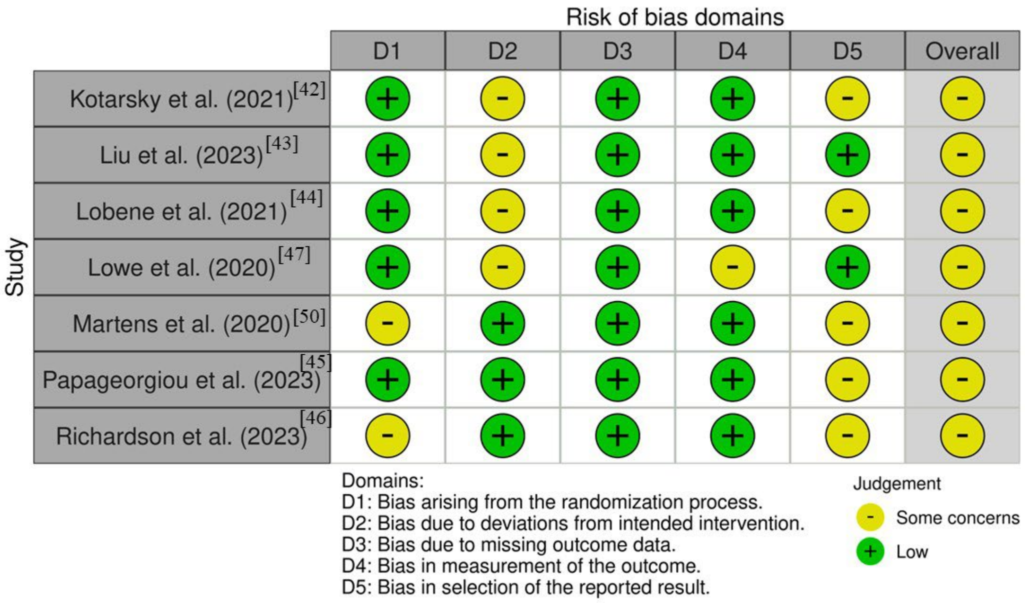

Supplement: Supplementary file 1 [file nutrients-16-00876-s001.zip › nutrients-2880503-supplementary.pdf]
